# Role of the PB2 627 Domain in Influenza A Virus Polymerase Function

**DOI:** 10.1128/JVI.02467-16

**Published:** 2017-03-13

**Authors:** Benjamin E. Nilsson, Aartjan J. W. te Velthuis, Ervin Fodor

**Affiliations:** a Sir William Dunn School of Pathology, University of Oxford, Oxford, United Kingdom; b Clarendon Laboratory, Department of Physics, University of Oxford, Oxford, United Kingdom; St. Jude Children's Research Hospital

**Keywords:** 627 domain, PB2, RNA polymerases, influenza, replication, transcription

## Abstract

The RNA genome of influenza A viruses is transcribed and replicated by the viral RNA-dependent RNA polymerase, composed of the subunits PA, PB1, and PB2. High-resolution structural data revealed that the polymerase assembles into a central polymerase core and several auxiliary highly flexible, protruding domains. The auxiliary PB2 cap-binding and the PA endonuclease domains are both involved in cap snatching, but the role of the auxiliary PB2 627 domain, implicated in host range restriction of influenza A viruses, is still poorly understood. In this study, we used structure-guided truncations of the PB2 subunit to show that a PB2 subunit lacking the 627 domain accumulates in the cell nucleus and assembles into a heterotrimeric polymerase with PB1 and PA. Furthermore, we showed that a recombinant viral polymerase lacking the PB2 627 domain is able to carry out cap snatching, cap-dependent transcription initiation, and cap-independent ApG dinucleotide extension *in vitro*, indicating that the PB2 627 domain of the influenza virus RNA polymerase is not involved in core catalytic functions of the polymerase. However, in a cellular context, the 627 domain is essential for both transcription and replication. In particular, we showed that the PB2 627 domain is essential for the accumulation of the cRNA replicative intermediate in infected cells. Together, these results further our understanding of the role of the PB2 627 domain in transcription and replication of the influenza virus RNA genome.

**IMPORTANCE** Influenza A viruses are a major global health threat, not only causing disease in both humans and birds but also placing significant strains on economies worldwide. Avian influenza A virus polymerases typically do not function efficiently in mammalian hosts and require adaptive mutations to restore polymerase activity. These adaptations include mutations in the 627 domain of the PB2 subunit of the viral polymerase, but it still remains to be established how these mutations enable host adaptation on a molecular level. In this report, we characterize the role of the 627 domain in polymerase function and offer insights into the replication mechanism of influenza A viruses.

## INTRODUCTION

The influenza virus genome consists of eight single-stranded negative-sense RNA segments. These viral RNA (vRNA) segments are coated by nucleoprotein (NP) and bound at their conserved 5′ and 3′ ends by the viral RNA-dependent RNA polymerase, forming viral ribonucleoprotein (vRNP) complexes. The conserved 5′ and 3′ vRNA ends are also referred to as the vRNA promoter. The viral polymerase consists of the three subunits polymerase acidic (PA), polymerase basic 1 (PB1) and polymerase basic 2 (PB2) proteins. During infection, the viral polymerase transcribes vRNA into mRNA and replicates it through a cRNA replicative intermediate (1). Transcription involves “cap snatching,” in which cellular capped RNA is bound by the PB2 cap-binding domain and cleaved by the PA endonuclease domain of the viral polymerase 8 to 14 nucleotides (nt) downstream of the 5′ m^7^G cap (2–4). These short capped RNA fragments serve as primers for viral mRNA synthesis by the resident (*cis*-acting) polymerase in the vRNP. Replication of viral RNA is initiated *de novo* in a primer-independent manner. During the first step of replication, cRNA is synthesized and nascent cRNA molecules assemble into vRNP-like complementary ribonucleoprotein (cRNP) complexes with newly synthesized polymerase and NP (5). During the second step of replication, cRNA serves as the template for vRNA synthesis. Replication involves terminal initiation at residues 1 and 2 on the vRNA template but internal initiation at residues 4 and 5 on the cRNA template (6, 7). Internal initiation leads to the generation of an ApG dinucleotide that is used to prime full-length vRNA synthesis at residues 1 and 2 of the cRNA (6, 7). Replication requires a *trans*-acting or *trans*-activating polymerase in addition to the resident polymerase (5, 8).

**FIG 1:**
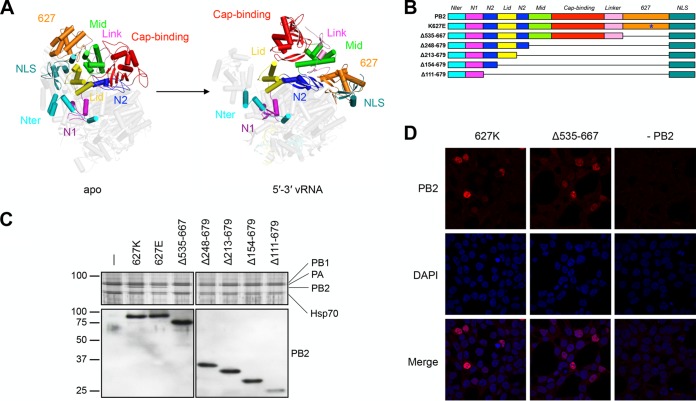
Design of PB2 deletion mutants and their expression. (A) Crystal structure of the (apo) influenza C/Johannesburg/1/66 virus polymerase (PDB code 5D98) and the (vRNA promoter bound) influenza A/little yellow-shouldered bat/Guatemala/060/10 (H17N10) virus polymerase (PDB code 4WSB) with the PB2 subunit domains shown in different colors. (B) Schematic of full-length, K627E, and truncated PB2 subunits with domains colored as in panel A. (C) Complex formation of K627E and PB2 truncation mutants with PB1 and PA. Polymerase subunits PB1 and PA were coexpressed with K627E or PB2 truncation mutants in HEK-293T cells and purified by IgG Sepharose chromatography. Purified recombinant polymerase was analyzed by SDS-PAGE. PA, PB1, and full-length PB2 were detected by silver staining. Copurification of the K627E mutant and truncated PB2 subunits with PB1 and PA was detected by Western blotting with a primary antibody targeting the N-terminal (positions 1 to 180) region of PB2 (36). Molecular masses, indicated on the left, are in kilodaltons. (D) Polymerase subunits PA, PB1, and PB2 lacking the 627 domain were coexpressed in HEK-293T cells, and PB2 was detected by immunofluorescence using a primary antibody targeting the N-terminal (positions 1 to 180) region of PB2 (36).

Crystal structures of the influenza virus RNA polymerase have shown that the enzyme consists of a central RNA polymerase domain, made up of PB1, the C-terminal domain of PA, the N-terminal one-third of PB2 (which includes the PB2 N terminus, the lid domain, and the N1 and N2 linker domains [Fig. 1A]), and several flexible peripheral appendices that are formed by the N-terminal PA endonuclease domain (2, 4) and the C-terminal two-thirds of PB2, including the cap-binding, mid-link, 627, and nuclear localization signal (NLS) domains (3, 9, 10). Two of the three major protruding peripheral domains, the cap-binding and endonuclease domains, are involved in the cap snatching process by binding to and cleaving host capped RNA, respectively, while the role of the so-called 627 domain in polymerase function remains unclear. Recently published crystal structures of influenza virus RNA polymerases have revealed that binding of the conserved vRNA or cRNA terminal ends can trigger significant conformational rearrangements in the peripheral domains (11–14) (Fig. 1A). The conformation that the polymerase adopts following vRNA binding, the open form, is compatible with transcription, while the more closed form is not. However, the closed form could be consistent with replication.

Avian influenza virus polymerases are severely restricted in their activity in mammalian hosts, resulting in impaired viral genome replication and mRNA synthesis and a reduced potential for acquiring beneficial mutations (15). For instance, avian influenza A virus polymerases almost universally contain a glutamic acid (E) residue at position 627 of PB2, whereas this residue is frequently mutated to lysine (K) in mammal-adapted polymerases (16). It has been shown that changing the glutamic acid residue at position 627 of PB2 to lysine (E627K) restores activity of avian polymerases in mammalian cells (17–19). PB2 residue 627 is located on the 627 domain, which folds into a structurally distinct domain protruding from the polymerase core. In the conformation that the RNA polymerase adopts following the binding of vRNA, PB2 residue 627 is surface exposed and located near the presumed nascent RNA exit channel (9, 10, 12).

In addition to E627K, several other adaptive mutations have been found to cluster on the surface of the 627 domain or in the close vicinity of the 627 domain and the nascent RNA exit channel (20, 21). On the mammal-adapted PB2 surface, these residues form a basic groove, whereas in avian polymerases, acidic residues disrupt this basic patch (9, 10). The basic surface on the 627 domain of mammal-adapted influenza virus polymerases appears to be important for efficient viral polymerase activity and RNA binding in mammalian cells (22–24). Adaptive mutations are not needed in all of these host-specific sites, as some of them can compensate for the lack of others by individually enhancing avian polymerase activity in mammalian cells, which suggests a degree of redundancy among the adaptive mutations (25).

Although adaptive mutations have been demonstrated to enhance the activity of avian influenza virus polymerases in mammalian cells, there is disparity in the literature regarding the mechanism through which these mutations enhance polymerase activity. For instance, it has been proposed that 627E could impair vRNP assembly in mammalian cells by destabilizing polymerase and NP interactions (26–28), but this view has been challenged in recent studies (17, 19). The PB2 627 residue has also been proposed to regulate viral promoter binding (19, 29), interactions between the viral polymerase and importin-α (30, 31), the interaction between the virus polymerase and a cellular inhibitor of the virus infections in human cells (18), or the interaction between the polymerase and an activating host factor (32). In support of the last item, the cellular protein ANP32A was recently identified as an underlying factor in influenza A virus polymerase host restriction mediated by residue 627 of PB2 (33). However, no molecular mechanism has yet been presented that can explain how PB2 residue 627 determines host range.

In this study, using a combination of *in vitro* polymerase activity assays and cell-based vRNP reconstitution assays, we characterized the role of the flexible C-terminal two-thirds of PB2 in influenza virus polymerase activity. We report that the influenza virus polymerase has different requirements for PB2 domains, depending on whether it uses vRNA or cRNA as the template. We also show that the 627 domain of PB2 is not required for basic polymerase activities such as binding of viral RNA and transcription of viral RNA *in vitro* but that the domain is essential for viral RNA replication and transcription in a cellular context. Furthermore, we show that a replicating RNA polymerase that uses a vRNA as the template requires a second polymerase with a 627 domain to stabilize the nascent cRNA in a cellular context.

## RESULTS

### PB2 mutants with C-terminal truncations form stable complexes with the PB1 and PA dimer.

In order to address the function of the flexible C-terminal two-thirds of PB2 (Fig. 1A) and to identify the minimal PB2 region required for core polymerase functions, we generated pcDNA-PB2 constructs expressing PB2 of influenza A/WSN/33 (H1N1) virus with systematic internal truncations but a preserved C-terminal NLS (Fig. 1B). We first sought to determine whether these PB2 mutants could form a heterotrimeric complex with PB1 and PA and coexpressed them with PB1 and PA fused to a tandem affinity purification tag (PA-TAP) in human HEK-293T cells. A polymerase with a lysine-to-glutamic acid change at position 627 of PB2 (K627E), characteristic of avian influenza virus polymerases, was also included. Purification of the recombinant polymerases from cell lysates using the protein A tag on PA and analysis of the purified complexes by SDS-PAGE and silver staining showed that PB1 and PA formed a stable dimer in the absence of PB2, in agreement with previous findings (34, 35). Western blotting with an antibody raised against the N-terminal 180 amino acids of PB2 (36) revealed that all PB2 truncation mutants as well as the K627E mutant formed heterotrimer complexes with PB1 and PA (Fig. 1C).

It is believed that polymerase complex formation takes place in the nucleus after separate nuclear import of the PB1-PA dimer and the PB2 monomer (34, 37–39). To confirm that complex assembly had indeed occurred in the nucleus and that the deletion of the 627 domain had no impact on the subcellular localization of the influenza virus polymerase, we coexpressed polymerase subunits PA, PB1, and PB2 in HEK-293T cells and determined the localization of the PB2 subunit with or without the 627 domain using immunofluorescence. Confocal microscopy revealed a predominantly nuclear localization for both the wild-type PB2 polymerase subunit and the PB2 subunit lacking the 627 domain (Fig. 1D). Together, these results show that the PB2 lid, cap-binding, mid-link, and 627 domains are not required for stable complex formation with PA and PB1 and that the lack of a 627 domain does not affect the nuclear accumulation of PB2.

### Polymerase lacking the 627 domain of PB2 is active *in vitro*.

**FIG 2:**
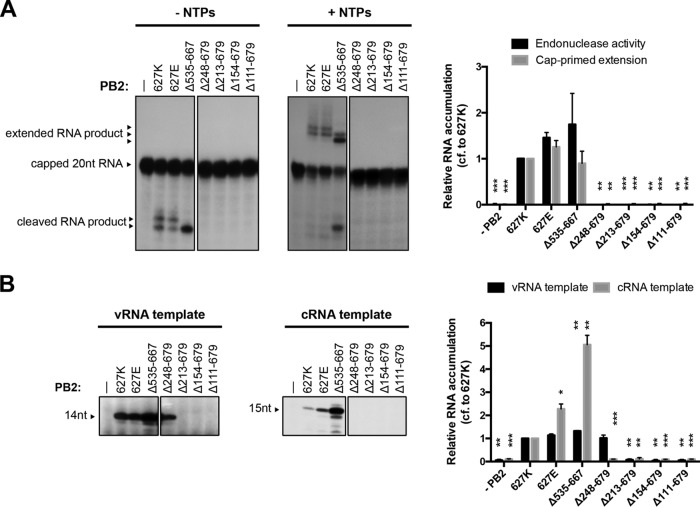
Polymerase lacking the PB2 627 domain is active *in vitro*. Purified recombinant polymerases were incubated in reaction mixtures containing radiolabeled capped RNA and vRNA template in the absence (left) or presence (right) of nucleotides (A) or ATP, UTP, CTP, [α-^32^P]GTP, ApG primer, and either vRNA or cRNA as the template (B). Reaction products were analyzed by 20% PAGE and autoradiography. The graphs show the mean intensity signal relative to that of wild-type polymerase with K627 PB2 from three independent biological replicates (*n* = 3), with error bars representing the standard errors of the means and the asterisks indicating a significant difference from 627K (two-tailed one-sample *t* test) as follows: *, *P* < 0.05; **, *P* < 0.01; and ***, *P* < 0.001.

In order to address the question of whether the PB2 lid, cap-binding, mid-link, and 627 domains are needed for the initiation of RNA synthesis, we performed *in vitro* activity assays using purified recombinant polymerase. First, we evaluated the ability of the polymerases with PB2 mutations to carry out the cap snatching and capped RNA primer extension steps that are required of viral transcription. Purified polymerase was bound to vRNA promoter, comprising a 15-nt-long RNA corresponding to the 5′ end of vRNA and a 14-nt-long RNA corresponding to the 3′ end of vRNA, and incubated with a 20-nt-long radiolabeled capped RNA in the absence (to assess cap snatching) or presence (to assess capped RNA priming) of nucleotides. In the absence of nucleotides, the wild-type and K627E mutant polymerases produced two major cleavage products (Fig. 2A, left). These major cleavage products were extended into two major transcription products in the presence of nucleotides (Fig. 2A, right). The polymerase lacking the 627 domain (Δ535-667) was also active in cap snatching but produced only a single cleavage product and two extension products that were produced in unequal amounts. Moreover, the major extension product was shorter than the products of the wild-type and K627E polymerases. This suggests that the 627 domain might affect the relative positions of the cap-binding and endonuclease domains of the polymerase and that a deletion of the 627 domain results in cleavage and initiation taking place at alternative nucleotides of the capped RNA primer and vRNA template, respectively. Deletions of additional domains of PB2 resulted in polymerases inactive in cap snatching, which is in line with the fact that these all lack the cap-binding domain.

Next, we analyzed the ability of the polymerases to initiate viral replication, using the extension of an ApG dinucleotide primer on vRNA or cRNA templates as a readout (Fig. 2B). Polymerase was bound to vRNA promoter (see above) or cRNA promoter, which comprises a 14-nt-long RNA corresponding to the 5′ end of cRNA and a 15-nt-long RNA corresponding to the 3′ end of cRNA, and incubated in the presence of ApG and nucleotides. The wild-type polymerase, K627E polymerase, and the polymerase lacking the 627 domain (Δ535-667) were all able to elongate an ApG primer, producing 14- and 15-nt-long products on the vRNA and cRNA templates, respectively. The polymerase lacking the 627, cap-binding, and mid-link domains (Δ248-676) was also able to elongate ApG on the vRNA template but was unable to elongate ApG on the cRNA template. Further deletions into the nonflexible N-terminal third of PB2 resulted in polymerases incapable of ApG elongation on either template. Interestingly, the polymerase lacking the 627 domain exhibited an increased ApG extension on both templates (Fig. 2B).

Taken together, these results show that a polymerase lacking the 627 domain is active in cap snatching, cap-dependent transcription initiation, and ApG extension *in vitro*, indicating that the 627 domain is not required for core polymerase functions. Furthermore, our data show that a polymerase lacking not only the 627 domain but also the cap-binding and mid-link domains (Δ248-676) is active on the vRNA but not the cRNA template, revealing differential requirements of the PB2 structure for viral transcription and replication initiation on the vRNA and cRNA promoters.

### The 627 domain is required for polymerase activity in cells.

**FIG 3:**
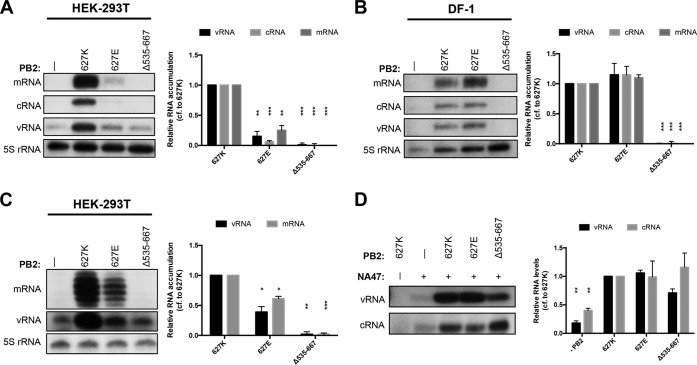
The PB2 627 domain is required for polymerase activity in the cell. Human HEK-293T (A) or chicken DF-1 (B) cells were cotransfected with plasmids expressing PA, PB1, wild-type or mutant PB2, NP, and segment 6 vRNA. Human HEK-293T cells (C) were cotransfected with plasmids expressing PA, PB1, wild-type or mutant PB2, and a 47-nt-long segment 6-derived vRNA. Accumulation of mRNA, cRNA, and vRNA was assessed by primer extension. The graphs show the mean intensity signal (with the mean intensity signal with no PB2 expressed subtracted) relative to that of wild-type PB2 627K polymerase from three independent biological replicates (*n* = 3), with error bars representing the standard errors of the means and the asterisks indicating a significant difference from the wild type (two-tailed one-sample *t* test) as follows: *, *P* < 0.05; **, *P* < 0.01; and ***, *P* < 0.001. (D) Polymerase subunits PA-TAP, PB1 with an active-site mutation (PB1a), wild-type and mutant PB2, and segment 6-derived 47-nt-long vRNA or cRNA were coexpressed in HEK-293T cells and purified by IgG Sepharose chromatography. Levels of vRNA or cRNA that copurified with polymerase were assessed by primer extension. The graphs show the mean intensity signal (with the mean intensity signal with no RNA expressed subtracted) relative to that of wild-type 627K polymerase from three independent biological replicates (*n* = 3), with error bars representing the standard errors of the means and the asterisks indicating a significant difference from 627K (two-tailed one-sample *t* test) as follows: *, *P* < 0.05; **, *P* < 0.01; and ***, *P* < 0.001.

Having shown that a polymerase lacking the 627 domain is active in transcription and ApG extension *in vitro*, we next evaluated the role of the 627 domain in polymerase activity in a cellular context using a mini-replicon assay. RNPs were reconstituted by coexpression of the three polymerase subunits, NP, and segment 6 vRNA in human HEK-293T cells, and the accumulation of positive- and negative-sense viral RNAs was analyzed by primer extension. In contrast to results obtained *in vitro* (Fig. 2), but in agreement with previous findings (18, 19), the “avian-like” K627E polymerase was significantly restricted in its ability to transcribe and replicate vRNA compared to the “mammalian-like” wild-type polymerase (627K) (Fig. 3A). Furthermore, the polymerase lacking the 627 domain (Δ535-667) was unable to generate viral RNAs. To address the question of whether the PB2 627 domain is also required for activity in avian cells, RNPs were reconstituted in avian DF-1 cells. The wild-type mammalian-like polymerase and the K627E avian-like polymerase showed similar levels of RNA accumulation, whereas the mutant lacking the 627 domain was not able to replicate or transcribe vRNA (Fig. 3B).

Position 627 of PB2 has been implicated in RNP assembly, putatively by recruiting or interacting with NP (26–28). To investigate whether the inability to synthesize vRNA in cells of the polymerase lacking the 627 domain is associated with a disruption of polymerase-NP interaction, we replaced the segment 6 vRNA with a 47-nt-long vRNA that can be transcribed and replicated in an NP-independent manner (40). Primer extension analysis showed significant levels of RNA accumulation by the avian-like 627E polymerase (Fig. 3C), in agreement with previous findings that the 627E-mediated restriction is diminished on short vRNA templates in mammalian cells (19). Deletion of the 627 domain was also detrimental to polymerase activity on the short template. This suggests that a disruption of polymerase-NP interactions is not the primary cause for the lack of RNA synthesis by the 627 domain deletion mutant. However, we cannot exclude the possibility that the 627 domain is also required for RNP assembly on longer templates by being involved in polymerase-NP interactions.

Next we tested the ability of the polymerase lacking the 627 domain to bind vRNA and cRNA in the cell. Recombinant polymerase with an active-site mutation in PB1 (D445A/D446A), which allows RNA binding but no transcription or replication (41), was coexpressed with a 47-nt-long vRNA or cRNA and purified via a protein A tag on PA. RNA that was bound by these purified polymerases was subsequently extracted and analyzed by primer extension. While only low levels of copurifying vRNA and cRNA could be detected for a PA-PB1 dimer control, in line with previous observations that the PB2 submit is required for efficient promoter binding (35), the copurifying vRNA and cRNA levels were similar with the wild-type, K627E, and 627 domain deletion mutant polymerases (Fig. 3D). This, together with the previous *in vitro* activity data, shows that a polymerase lacking the 627 domain is able to bind to viral RNA.

Overall, our data show that the 627 domain is essential for the transcription and replication of vRNA in a cellular context, independent of the host species and the requirement for NP. However, the mutant lacking the 627 domain is able to bind vRNA and cRNA. Furthermore, these data confirm previous findings that the amino acid at position 627 of PB2 plays a central role in determining the ability of the polymerase to transcribe and replicate vRNA in a mammalian host.

### The PB2 627 domain is required for the accumulation of cRNA in infected cells.

**FIG 4:**
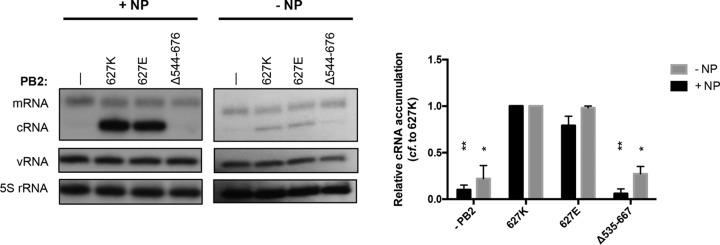
The PB2 627 domain is required for cRNA accumulation in infected cells. PA, PB1, and wild-type or mutant PB2 were coexpressed in HEK-293T cells in the presence (left) or absence (right) of NP. Twenty-four hours posttransfection, cells were infected with influenza A/WSN/33 virus at an MOI of 10 in the presence of actinomycin D. Six hours postinfection, total RNA was extracted and analyzed by primer extension. The graphs show the mean intensity signal relative to that of wild-type 627K polymerase from three independent biological replicates (*n* = 3), with error bars representing the standard errors of the means and the asterisks indicating a significant difference from 627K (two-tailed one-sample *t* test) as follows: *, *P* < 0.05, and **, *P* < 0.01.

The findings described above show that the PB2 627 domain is not required for core polymerase functions *in vitro* but that it is essential for the accumulation of viral RNAs in the cell. In a cellular context, free polymerase needs to be recruited to replicating vRNPs to stabilize newly synthesized nascent cRNA and protect it from host nucleases (41). In order to investigate whether a polymerase lacking the 627 domain can stabilize cRNA in the cell, the PB1 active-site mutant, which binds but does not replicate or transcribe vRNA, was preexpressed in combination with PA and wild-type or mutant PB2 in human HEK-293T cells prior to an infection of the transfected cells with influenza A/WSN/33 (H1N1) virus in the presence of actinomycin D, an inhibitor of cellular transcription. Total RNA was isolated and viral RNAs were analyzed by primer extension. As shown in Fig. 4, mammalian-like wild-type (627K) and avian-like 627E polymerases stabilized cRNA equally well, whereas a PA-PB1 dimer and polymerase lacking the 627 domain were unable to stabilize cRNA.

Taken together, these results show that the PB2 627 domain is required for the stabilization of cRNA in infected cells. However, the nature of amino acid at position 627 of PB2 does not affect the ability of the polymerase to stabilize cRNA.

## DISCUSSION

In this study, we aimed to gain insight into the function of the C-terminal domains of PB2 and in particular the 627 domain of the influenza A virus RNA polymerase. We found that the PB2 627 domain is not required for the nuclear accumulation of PB2 and polymerase heterotrimer assembly. A recombinant polymerase lacking the 627 domain is able to carry out core polymerase functions such as capped RNA primer-dependent transcription initiation and RNA synthesis *in vitro*. However, in a cellular context, it is unable to replicate viral RNA, although it can still bind vRNA and cRNA templates. Furthermore, vRNPs do not produce cRNA in infected cells if polymerase lacking the PB2 627 domain is provided in *trans*, suggesting that the 627 domain may play a role in viral RNA genome replication through mediating polymerase association.

The finding that PB2 627 domain is not required for nuclear accumulation and polymerase assembly is consistent with previous data. PB2 is imported into the nucleus through its C-terminal NLS using the classical importin-α/β nuclear import pathway (42). Our data suggest that deletion of the 627 domain does not affect the interaction of the C-terminal NLS with importin-α. Furthermore, the finding that the 627 domain is not required for polymerase heterotrimer assembly is in agreement with previous findings that N-terminal PB2 fragments can form stable complexes with the PB1-PA dimer (35, 43).

Our data show that a polymerase lacking the 627 domain is capable of RNA synthesis *in vitro*, which indicates that the 627 domain does not contribute to the core polymerase functions. This is consistent with the location of the 627 domain on the outside of the polymerase core, which is made up of PB1, the C-terminal domain of PA, and an N-terminal third of PB2 (11–13). The 627 domain is also not required for RNA template binding, as the RNA-binding functions reside in the polymerase core (12, 13). The 627 domain-deficient polymerase was found to be able to cleave capped RNA and extend the resulting capped RNA primer. However, interestingly, it produced a shorter major capped RNA product in the extension reaction than the wild-type polymerase. This suggests that the lack of the 627 domain affects the positioning of the cap-binding domain and the polymerase active site and that the 627 domain plays a minor structural role in influenza virus transcription.

Our study also revealed a differential requirement for the PB2 domains N terminal of the 627 domain in ApG-primed RNA synthesis on vRNA and cRNA templates. While a 627 domain-deficient polymerase could extend ApG on both vRNA and cRNA templates, deletion of the 627, cap-binding, and mid-link domains resulted in a polymerase that was able to extend ApG on a vRNA but not on a cRNA template. The differential requirement of PB2 domains for these polymerase activities could be linked to the different modes of initiation of the polymerase. While the initiation of replication on the vRNA template occurs at positions 1 and 2, initiation on the cRNA template takes place at positions 4 and 5, resulting in a pppApG dinucleotide that is used as a primer for full-length vRNA synthesis after backtracking of the cRNA template in the polymerase active site (6). Furthermore, initiation on the vRNA template requires a priming loop, a β-hairpin protruding into the polymerase active site, but for initiation on the cRNA template, the priming loop needs to retract (7). We thus speculate that deletion of the mid-link and cap-binding domains might affect the ability of the template to backtrack or the priming loop to retract, leading to the inhibition on the cRNA but not on the vRNA template. Further deletions of PB2 resulted in polymerases with no detectable activity in RNA synthesis, demonstrating that the N-terminal third of PB2 (amino acids 1 to 247) is not only structurally but also functionally a part of the polymerase core.

Although the 627 domain is not important for RNA polymerase activity *in vitro*, it is absolutely essential for viral RNA accumulation in RNP reconstitution assays in a cellular context. Previous studies suggested that the nature of the amino acid at position 627 affects the assembly of viral RNPs (26–28). However, we found that deletion of the 627 domain also prevents replication of short vRNA templates that can be replicated in the absence of NP (40). This suggests that the inhibition of polymerase function by the deletion of the 627 domain is not simply due to a disruption of an interaction between the polymerase and NP.

How does, then, the PB2 627 domain contribute to viral RNA replication in a cellular context? We found that vRNPs do not produce cRNA in infected cells if the polymerase lacking the PB2 627 domain is provided in *trans*. Previous studies suggested that vRNPs produce cRNA early in infection but it gets degraded unless polymerase and NP reach sufficient levels to bind and stabilize it (41). However, these studies did not exclude the possibility that, in a cellular context, the stabilizing polymerase also needs to associate with the replicating polymerase and possibly *trans*-activate it before cRNA is produced. We found that vRNA and cRNA expressed through plasmid transfection could be copurified with the polymerase lacking the 627 domain, suggesting that RNA binding is not affected, in agreement with the *in vitro* data described above. If the 627 domain-deficient polymerase can bind cRNA, it follows that it must be deficient in binding and/or *trans*-activating the vRNP-resident polymerase. Indeed, oligomerization of the polymerase heterotrimer has been proposed (35, 43, 44), and a *trans*-activating or a *trans*-acting polymerase has been implicated in replication (5, 8). It is tempting to speculate that the PB2 627 domain might be involved in the interaction between the RNP-resident and the *trans*-acting or *trans*-activating polymerase; alternatively, the 627 domain might be involved in the interaction with a cellular factor that could be important for the recruitment of the *trans*-acting or *trans*-activating polymerase to RNPs. Recently, cellular ANP32A has been identified as a host factor underlying the host restriction mediated by PB2 amino acid residue 627 (33). Avian-like (PB2-627E) and mammalian-like (PB2-627K) polymerases are equally able to stabilize cRNA, suggesting that while the 627 domain as a whole might be important for polymerase recruitment, the nature of position 627 itself does not affect this process.

In summary, we demonstrate here that the PB2 627 domain of the influenza virus RNA polymerase is not involved in core catalytic functions of the polymerase but that it is essential for the replication of RNPs in a cellular context. We propose that the 627 domain in RNA-free polymerases is necessary for recruitment of the polymerase to replicating vRNPs. These findings further our understanding the role of the PB2 627 domain in transcription and replication of the influenza virus RNA genome by the viral polymerase.

## MATERIALS AND METHODS

### Cells, viruses, and plasmids.

Human embryonic kidney 293T cells (HEK-293T) and chicken embryonic fibroblasts (DF-1) were cultured in Dulbecco modified Eagle medium (DMEM) supplemented with 10% fetal calf serum (FCS). Madin-Darby bovine kidney (MDBK) epithelial cells were cultured in minimal essential medium (MEM) supplemented with 10% FCS and 2 mM l-glutamine. All cells were maintained at 37°C and 5% CO_2_. Recombinant wild-type influenza A/WSN/33 (H1N1) virus was generated using the pHW2000 eight-plasmid system (45). Plasmids pcDNA-NP, pcDNA-PA, pcDNA-PB1, pcDNA-PB2 (46), pcDNA-PA-TAP (38), pcDNA-PB1a (41), and pcDNA-PB2-627E (27), expressing influenza A/WSN/33 virus proteins, as well as the vRNA-expressing plasmids pPOLI-NA-RT (47) and pPOLI-NA47 and pPRC425-NA (19) have been described previously. Plasmid pcDNA3A has also been described previously (46). pcDNA-PB2 plasmids expressing PB2 with internal deletions were made by site-directed PCR mutagenesis. pPOLI-cNA47 was generated from pPOLI-cNA-RT (48) as described for pPOLI-NA47.

### Immunofluorescence microscopy.

HEK-293T cells grown on sterilized glass coverslips in 24-well plates were transiently transfected with 0.25 μg each of pcDNA-PA, pcDNA-PB1, and pcDNA-PB2/pcDNA-PB2Δ535-667/pcDNA3A using Lipofectamine 2000 reagent (Invitrogen) and OPTIMEM (Invitrogen) according to the manufacturer's instructions. Forty-eight hours posttransfection, cells were fixed for 15 min in 4% formaldehyde in 250 mM HEPES (pH 7.9) and permeabilized for 15 min in 0.25% Triton X-100 in phosphate-buffered saline (PBS). Cells were blocked in PBS containing 10% normal goat serum, 0.5% Triton X-100, and 3% bovine serum albumin overnight at 4°C. Cells were stained with a polyclonal rabbit antibody raised against the N-terminal 180 amino acids of PB2 (36) and an Alexa Fluor 532-conjugated anti-rabbit secondary antibody (Thermo Fisher). Coverslips were mounted in Mowiol containing 4′,6-diamidino-2-phenylindole (DAPI), and images were obtained with an Olympus FV1000 confocal laser scanning microscope. Image analysis was carried out using ImageJ software (49).

### Tandem affinity purification of recombinant influenza virus polymerase.

HEK-293T cells were transiently transfected in 10-cm dishes using 5 μg each of pcDNA-PA-TAP, pcDNA-PB1, and pcDNA-PB2 (or PB2 mutant as indicated) using Lipofectamine 2000 reagent (Invitrogen) and OPTIMEM (Invitrogen) according to the manufacturer's instructions. Cells were harvested 48 h posttransfection, lysed in 500 μl of Tris lysis buffer (50 mM Tris-HCl [pH 8.0], 200 mM NaCl, 25% glycerol, 0.5% Igepal CA-630, 1 mM dithiothreitol [DTT], 1 mM phenylmethylsulfonyl fluoride [PMSF], 1× complete EDTA-free protease inhibitor cocktail tablet [Roche]) at 4°C for 1 h and centrifuged at 17,000 × *g* for 5 min. The cleared cell lysate was diluted 1:5 in binding buffer (20 mM Tris-HCl [pH 8.0], 150 mM NaCl) and incubated with washed IgG Sepharose (GE Healthcare) (50 μl per sample) at 4°C for 3 h. After binding, the IgG Sepharose beads were washed three times in wash buffer (10 mM Tris-HCl [pH 8.0], 150 mM NaCl, 10% glycerol, 0.1% Igepal CA-630, 1 mM PMSF). Recombinant polymerase was released using tobacco etch virus (AcTEV) protease in elution buffer (10 mM Tris-HCl [pH 8.0], 150 mM NaCl, 10% glycerol, 0.1% Igepal CA-630, 1 mM dithiothreitol, 1 mM PMSF, 1× complete EDTA-free protease inhibitor cocktail tablet) at 4°C overnight and cleared from the beads at 17,000 × *g* for 5 min. Purified polymerase was analyzed by SDS-PAGE and silver staining (SilverXpress; Invitrogen) as well as Western blotting using a polyclonal rabbit antibody raised against the N-terminal 180 amino acids of PB2 (36) as the primary antibody and a horseradish peroxidase (HRP)-conjugated goat anti-rabbit IgG as the secondary antibody (Sigma-Aldrich) and the Immobilon Western chemiluminescence HRP substrate kit (Millipore) for detection.

### *In vitro* capped-RNA cleavage and extension assays.

To analyze the capped-RNA cleavage and extension activity of the viral polymerase, first a synthetic 20-nucleotide RNA with 5′ diphosphate (5′-ppAAUCUAUAAUAGCAUUAUCC-3′) (Chemgenes) was capped with a radiolabeled cap 1 structure in 20-μl reaction mixtures containing 1 μM RNA, 0.25 μM [α-^32^P]GTP (3,000 Ci/mmol; Perkin-Elmer), 0.8 mM *S*-adenosylmethionine, 0.5 U/μl of vaccinia virus capping enzyme (New England BioLabs [NEB]), and 2.5 U/μl of 2′-*O*-methyltransferase (NEB) at 37°C for 1 h. The product was analyzed by 16% denaturing PAGE, excised, eluted overnight in deionized water, and desalted using NAP-10 columns (GE Healthcare). Capped-RNA cleavage assays were performed in a reaction mixture containing 5 mM MgCl_2_, 1 mM DTT, 2 U/μl of RNasin, 0.5 μM 5′ vRNA promoter, 0.5 μM 3′ vRNA promoter, ∼1,500 cpm of capped RNA, and 5 ng/μl of polymerase in 0.5× polymerase elution buffer. Extension assays were performed in a reaction mixture containing 5 mM MgCl_2_, 1 mM DTT, 2 U/μl of RNasin, 0.5 μM 5′ vRNA promoter, 0.5 μM 3′ vRNA promoter, 1 mM ATP, 0.5 mM UTP, 0.5 mM CTP, 0.5 mM GTP, ∼1,500 cpm of capped RNA primer, and 5 ng/μl of polymerase in 0.5× polymerase elution buffer. Reaction mixtures were incubated at 30°C for 4 h and stopped by addition of an equal volume of 80% formamide, 1 mM EDTA, and bromophenol blue and xylene cyan dyes and incubation at 95°C for 3 min. Reaction products were resolved by 20% denaturing PAGE containing 7 M urea in Tris-borate-EDTA (TBE) buffer and visualized by autoradiography. ImageJ was used to analyze the ^32^P-derived signal (49).

### *In vitro* ApG extension assay.

The ability of purified polymerase to extend an ApG dinucleotide was tested as described previously (6). Reaction mixtures containing 1 mM ATP, 0.5 mM CTP, 0.5 mM UTP, 0.25 mM ApG, 5 mM MgCl_2_, 1 mM DTT, 2U/μl of RNasin, 0.05 μM [α-^32^P]GTP (3,000 Ci/mmol; Perkin-Elmer), 0.5 μM 5′ vRNA (or cRNA), 0.5 μM 3′ vRNA (or cRNA), and 5 ng/μl of polymerase in 0.5× polymerase elution buffer were incubated at 30°C for 1 to 4 h. Reactions were stopped by adding an equal volume of 80% formamide, 1 mM EDTA, and bromophenol blue and xylene cyan dyes and incubation at 95°C for 3 min. Reaction products were resolved by 20% denaturing PAGE containing 7 M urea in TBE buffer and visualized by autoradiography. ImageJ was used to analyze the ^32^P-derived signal (49).

### RNP reconstitution and primer extension analysis.

HEK-293T or DF-1 cells in DMEM supplemented with 10% FCS were transiently transfected in 35-mm dishes using Lipofectamine 2000 (Invitrogen) and OPTIMEM (Invitrogen) according to the manufacturer's instructions. One microgram each of pcDNA-PA, pcDNA-PB1, pcDNA-PB2, and pcDNA-NP as well as a vRNA-expressing plasmid (pPOLI-NA-RT or pPRC425-NA for full-length neuraminidase [NA] segment vRNA template or pPOLI-NA47 for a short [47-nt] vRNA template) was transfected. Cells were harvested 48 h posttransfection. Total RNA was extracted using TRI Reagent (Sigma-Aldrich) and dissolved in 10 μl of double-distilled water (ddH_2_O). The accumulation of viral mRNA, vRNA, and cRNA was analyzed by primer extension using ^32^P-labeled primers specific for negative- or positive-sense NA RNA (50) and negative- or positive-sense NA47 RNA (19). Primer extension products were analyzed by 6 to 14% denaturing PAGE with 7 M urea in TBE buffer and detected by autoradiography. ImageJ was used to analyze the ^32^P-derived signal (49). Signal levels were adjusted to 5S rRNA, which was used as an internal control.

### RNA binding assay.

HEK-293T cells in DMEM supplemented with 10% FCS were transiently transfected in 10-cm dishes with 5 μg of pcDNA-PA-TAP, pcDNA-PB1a, pcDNA-PB2, and pPOLI-NA47 or pPOLI-cNA47 using Lipofectamine 2000 (Invitrogen) and OPTIMEM (Invitrogen) according to the manufacturer's instructions. Recombinant polymerase was purified after 48 h as described above. RNA was extracted from purified polymerase using TRI Reagent (Sigma-Aldrich) and dissolved in 10 μl of ddH_2_O. The presence of RNA bound to polymerase was analyzed by primer extension using^32^P-labeled primers specific for negative- or positive-sense NA47 RNA (19). Primer extension products were analyzed by 14% denaturing PAGE with 7 M urea in TBE buffer and detected by autoradiography. ImageJ was used to analyze the ^32^P-derived signal (49).

### cRNA stabilization assay.

293T cells were transiently transfected in 35-mm dishes as described above with 1 μg each of pcDNA-PA, pcDNA-PB1a, and pcDNA-PB2 with or without 2 μg of pcDNA-NP. Twenty-four hours posttransfection, cells were infected with influenza A/WSN/33 virus at a multiplicity of infection (MOI) of 10 in the presence of 10 μg/ml of actinomycin D. Total RNA was extracted 6 h postinfection and analyzed by primer extension as described above.
